# Incremental Learning in Modelling Process Analysis Technology (PAT)—An Important Tool in the Measuring and Control Circuit on the Way to the Smart Factory

**DOI:** 10.3390/s21093144

**Published:** 2021-05-01

**Authors:** Shivani Choudhary, Deborah Herdt, Erik Spoor, José Fernando García Molina, Marcel Nachtmann, Matthias Rädle

**Affiliations:** 1 Center for Mass Spectrometry and Optical Spectroscopy, Mannheim University of Applied Sciences, Paul-Wittsack-Straße 10, 68163 Mannheim, Germany; e.spoor@hs-mannheim.de (E.S.); m.nachtmann@hs-mannheim.de (M.N.); m.raedle@hs-mannheim.de (M.R.); 2 Institute of Process Control and Innovative Energy Conversion, Mannheim University of Applied Sciences, 68163 Mannheim, Germany; Jose.Fernando.Garcia.Molina@continental-corporation.com

**Keywords:** SVM, incremental learning, Raman spectroscopy, process technology

## Abstract

To meet the demands of the chemical and pharmaceutical process industry for a combination of high measurement accuracy, product selectivity, and low cost of ownership, the existing measurement and evaluation methods have to be further developed. This paper demonstrates the attempt to combine future Raman photometers with promising evaluation methods. As part of the investigations presented here, a new and easy-to-use evaluation method based on a self-learning algorithm is presented. This method can be applied to various measurement methods and is carried out here using an example of a Raman spectrometer system and an alcohol-water mixture as demonstration fluid. The spectra’s chosen bands can be later transformed to low priced and even more robust Raman photometers. The evaluation method gives more precise results than the evaluation through classical methods like one primarily used in the software package Unscrambler. This technique increases the accuracy of detection and proves the concept of Raman process monitoring for determining concentrations. In the example of alcohol/water, the computation time is less, and it can be applied to continuous column monitoring.

## 1. Introduction

Process analysis technology (PAT) is established in many chemical industry plants. It enables the production of the required technical quality in compliance with safety standards. This is made possible with the best possible use of raw materials, systems, and energy. The chemical industry is the most energy-intensive manufacturing industry in Germany, with consumption of 1137.3 petajoules in 2018 [1]. This corresponds to a work or energy of 36.06 gigawatt years. From an economic perspective alone, resource efficiency is of great importance. From 1990 to 2018, the chemical–pharmaceutical industry was able to increase its production by 76%, reduce energy consumption by 17%, and reduce greenhouse gas emissions by 51% [2]. It is of the utmost importance to use sensors that significantly increase process understanding and allow more profound insight into the process. The profit is immense, especially for existing systems. Direct in-line measurement of the current composition for regulation is currently rarely used due to the high costs. Therefore, the typical sensors in-process application focus on process control variables—such as pressure and temperature—instead of in-line-product variables. However, measuring the composition of substances is particularly valuable to optimize processes economically and energetically. Optical processes are growing slowly because knowledge is obtained in-line and no extractions of the process have to be made, but accuracy is too low and the price too high. These systems must be flexible and quick to use to understand the process further. Permanent measuring points must be characterized by high measuring accuracy and reliability with low installation and operating costs. To guarantee process stability and, therefore, a high performance, the process has to be monitored. With high measurement density, digital output of the data, and numerous individual and real-time measurements, the system’s state becomes more predictable. Additionally, simultaneous measurements of several indicators of the process stabilize the predicted status, and consequently, rapid intervention, if necessary, is ensured.

A possible and suitable method for those mentioned challenges is Raman technology with high selectivity and detection sensitivity. The integration into the process can be realized with a flange connector mounted on an inspection glass in a pipe. The excitation wavelength from the laser and the scattered light from the sample can pass through the window [3,4]. With a calibration model, the resulting Raman shift intensities can be converted into the substance’s concentration. This continuous measurement variable leads to sustainable and safe controlled operations. From an economic point of view, the plant’s productivity can be increased or decreased as required. Therefore, this method can be adjusted according to demand, changing energy and raw material prices, leading to enhanced profit. Production efficiency is an increasingly differentiating characteristic for companies. This characteristic is per the sustainability requirements. Worldwide, there is a demand for conserving resources, reducing global warming gases, and, therefore, sustainable energy usage.

In the present work, an incremental learning evaluation model using a Support Vector Machine (SVM) model for Raman spectroscopic data is presented, which ensures user-friendly recalibration during operation. Its performance is determined experimentally and compared with conventional modelling techniques. SVM are robust classifiers, but large datasets lead to long computation times, high memory requirements, and increased complexity of the model. To solve this issue, SVM ensembles, where each SVM sees only a fraction of the data, are a viable solution [5]. Standard methods are used to achieve a high accuracy classifier by computing the best hyperparameters for the SVM model like tenfold cross-validation and grid search [6,7].

Generally, the studies using SVM learning from new data involve discarding the existing classifier, integrating the new data to the old set, and training a new classifier from scratch. The studies do not learn incrementally with the addition of new data, and they result in unlearning of data [5,8]. This means that the system cannot learn new information without forgetting previously learned classifiers. Such a problem is solved with the help of an incremental learning algorithm, defined as one that meets the following criteria [9,10]:

1. Can learn additional information from new data
2. Does not require access to the original data used to train the existing classifier
3. Preserves previously acquired knowledge

Additionally, the proposed incremental learning systems [11,12,13,14] suffer from high computation time and complexity. This framework’s main contribution is the implementation and evaluation of an incremental learning algorithm based on Garcia et al. [15], which uses parallel computing and helps reduce the computation time with reduced complexity as opposed to previously used learning algorithms [16]. The developed method’s relevance in the industrial environment is represented and discussed from a technical perspective. In the example process of rectification, a feed stream of ethanol and water is thermally separated, and ethanol is removed overhead as a distillate. Water leaves the column via the sump stream.

## 2. Materials and Methods

### 2.1. Experimental Setup Raman-Spectroscopy

**Figure 1:**
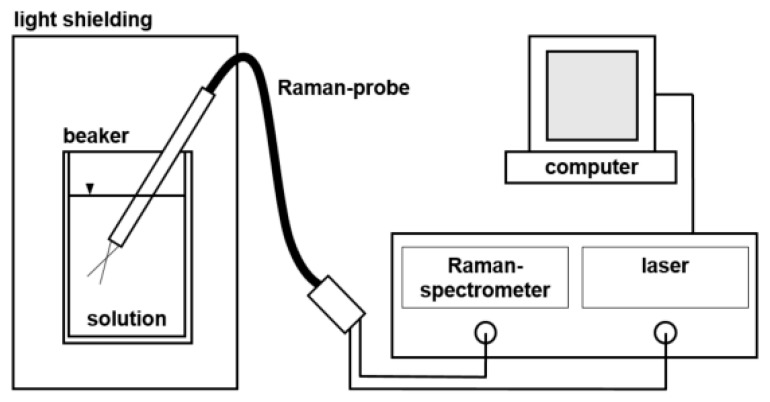
Experimental setup of Raman measurement.

Raman Spectroscopy was performed with a tec5 MultiSpec^®^Raman system (tec5, Steinbach, Germany), equipped with a coaxial designed RamanProbe II (InPhotonics, Norwood, Massachusetts, USA;fibre configuration: 105 µm excitation fibre; 600 µm collection fibre/working distance 7.5 mm). Raman scattering was excited by a 500 mW temperature-stabilized semiconductor laser (Raman Boxx™, PD-LD500) source at 785 nm. The Raman spectra were collected with a 1 cm^−1^ solution ranging from 300–3215 cm^−1^ using MultiSpec Pro II Raman process software (v1.4.1189.1826, tec5, Steinbach, Germany). The probe was clamped with a laboratory stand in the 50 mL borosilicate beaker (from Schott, Mainz, Germany). Figure 1 shows the setup schematically. Each solution (1–10) was measured ten times with an integration time of 10 s.

The dilution series was prepared with 96.2 Vol% Ethanol from VWR (Darmstadt, Germany, CAS No. 64-17-5) and distilled water. In total, ten solutions were prepared in 50 mL volumetric flasks (from Schott, Mainz, Germany). Initially, ethanol was transferred, with an Eppendorf Reference pipette (100-1000µL; Eppendorf AG, Hamburg, Germany), into the volumetric flask, and consequently, the remaining volume was filled up with distilled water according to Table 1. Solutions were then transferred into a 50 mL beaker for subsequent measurement.

### 2.2. Algorithm

In this work, the developed data evaluation method’s advantages were presented, which ultimately resulted in lowering the detection limit and increased robustness against outliers or poorly representative data. As a method, a learning algorithm was developed that has particular advantages concerning the learning speed in the continuous expansion of the database when new data were added. This was achieved by taking samples during monitoring as well as analyzing them in the laboratory. In time, they can be integrated into the model. The model improved continuously with accumulating data, which resulted in increased accuracy and a decreased error rate. The mathematical model used in this paper was adapted from the works of Garcia et al. [15].

Current computer-aided tools have made strides towards accurate and efficient detection of chemical concentrations. However, a common drawback observed in these approaches was the use of models that suffer from unlearning [5,8]. In these models, the previously learned knowledge was discarded, and new models had to be trained from scratch in the learning phase as soon as new data became available. In actual process conditions, in which the trained data were presented with time delay over a period of time, the standard gold data had a lack of stability. This was due to the sample taking influences, inhomogeneous product distribution, and impurities. This gave the reason that new emerging areas in machine learning systems must be investigated.

The advantage of an incremental learning algorithm was that it could learn additional information step by step as soon as new data were available. The ensemble learning algorithm used was implemented based on the work of Robi Polikar et al. [9]. The data set was divided into two classes: The first class corresponded to the spectra with a dilution greater than or equal to a specific dilution limit, and the second class contained the dilutions below this limit.

### 2.3. Mathematics

Based on the dynamically updated distribution of the training data set, the ensemble classifier was trained so that the samples that were more difficult to classify were given an increased probability of increasing their chances of being selected in the following training data set. The algorithm used the database $DB_{k},k=1,\ldots \ldots .,K$ where K represents the number of available measurement series, in this work ten. The random samples of all measurement series were first permuted arbitrarily and divided into K=10 stacks of equal size.

The inevitable case wording of SVM was used as the primary classifier, which is referred to below as 1C-SVM. The goal of training with the 1C-SVM was to establish an optimal hypothesis *h* with which the two classes were separated. The distance between the dividing line or hypothesis and the training data (called support vectors) of each class was maximized, and thus the model was protected against incorrect specifications and increased the robustness of the forecast. The goal was to determine an optimal hyperplane $f\left(x\right)=\varphi {\left(x_{i}\right)}^{T}w+w_{0}$ in the feature space. The following formula was used for this:

(1) $$min_{w,w_{0},\varepsilon }\frac{1}{2}{\left\|w\right\|}^{2}+C\overset{N}{\underset{i-1}{\sum }}\varepsilon_{i}$$

$$\mathrm{Subject}\;\mathrm{to}\;\varepsilon_{i}\geq 0\;y_{i}\left(\varphi {\left(x_{i}\right)}^{T}w+w_{0}\right)\geq 1-\varepsilon_{i\;}\forall i$$

In it forms $\phi \left(x_{i}\right)$ maps $\;x_{i}$ in a higher-dimensional space. w is the weight vector. C is a regulated hyperparameter that added a penalty to the target function in the event of overfitting. ε is a slip variable to weaken the limits and is called an error range or misclassification error. The Equation (1) can be determined using a quadratic approach with a second kind Lagrangian function [17]. By using this function, the kernel trick $\;K(x_{i},x_{j})$ can be used. With this it was possible to transfer the data to a higher dimension so that non-linear dependencies between different training data can be considered. The mapping in a higher dimensionality enabled the detection of similarities between data characteristics. The Gaussian radial basis function was used as the kernel, and accordingly, the following Equation (2) was obtained:

(2) $$K\left(x_{i},x_{j}\right)=exp\left(-\gamma \|x_{i}-x_{j}\|{}^{2}\right),\gamma =\frac{1}{2\sigma^{2}}$$

σ is the variance. γ is a hyperparameter that smoothed the kernel function, and this means a stronger or weaker relationship between the samples can be found depending on the γ values. To estimate the optimal hyperparameters γ and C for 1C-SVM, Nelder-Mead’s heuristic method was used [18,19]. The selected criterion to find the optimal classifier was the area under the Receiver Operating Characteristic curve (AUC-ROC), which is increasingly used in machine learning and evaluating more significant amounts of data.

The inputs for the ensemble algorithm were:

1. Training data $S_{k}=\{\left(x_{i},y_{i}\right)|i=1,\ldots \ldots \ldots ,N_{k}.$ The data set consisted of $N_{k}$ training data points with $x_{i}\in R^{d}$, where *d* represents the number of dimensions and $y_{i}\in \left\{-1,1\right\},$ the associated class. The $N_{k}$ data points were randomly selected from the $k^{th}$ database ($DB_{k}$).
2. A primary classifier to generate hypothesis *h*. The classifier required that at least 50 percent of the training data record was classified correctly.
3. An integer $T_{k}$ that specified the number of iteration steps $t=1,2,\ldots \ldots .,T_{k}$ for each data set, with t∈N. The prediction error could be reduced sufficiently with $T_{k}$.

The ensemble algorithm started with the initialization of a series of weights $\alpha_{t}$ for the training data set $S_{k}$ and a distribution $D_{t}$ obtained from $\alpha_{t}$ [1]. According to $D_{t}$, $S_{k}$ was divided into two subsets, $TR_{t}$ for training and $TE_{t}$ for validating during the $t^{th}$ iteration of the algorithm. $D_{t}$ was initially defined uniformly without deductive information. At each iteration, the weights adjusted at iteration t−1 were divided by the sum of all $W_{t}-1$ to ensure a legitimate distribution, and a new $D_{t}$ was computed. Training and test subsets were drawn randomly according to $D_{t}$. A hypothesis $h_{t}$ was obtained as the $t^{th}$ classifier, whose error $\varepsilon_{t}$ (3) was computed on the entire data set $S_{k}{}_{}$ with:

(3) $$\varepsilon_{t}=\frac{\sum_{i:h_{t}\left(x_{i}\right)}D_{t}\cdot \left|y_{i}-h_{k}\left(x_{i}\right)\right|}{\sum_{i:h_{t}\left(x_{i}\right)}D_{t}}$$

$\varepsilon_{t}$ was required to be less than 0.5 to ensure a reasonable performance of $h_{t}$. If the condition was satisfied, $h_{t}$ was accepted, and the error was normalized to calculate the normalization error $\beta_{t}$ (4):

(4) $$\beta_{t}=\frac{\varepsilon_{t}}{\left(1-\varepsilon_{t}\right)},\;0<\beta_{t}<1$$

The current $h_{t}$ was discarded if the condition was not satisfied, and a new training subset was selected. All hypotheses generated so far were then combined using the weighted majority voting to obtain a composite one $H_{t}$ (5), which allowed efficient incremental learning capability when new classes were introduced. The hypothesis with good performance was awarded a higher voting weight [20].

(5) $$H_{t}=\arg\max_{y\in Y}\underset{t:h_{t}\left(x\right)=y}{\sum }\log\left(\frac{1}{\beta_{t}}\right)$$

The error of $H_{t}$ was computed with (6) and must have also been less than 0.5 to ensure a reasonable performance of $H_{t}$; otherwise, the algorithm discarded that one and returned to select a new $TR_{t}$.

(6) $$E_{t}=\frac{\sum_{i:H_{t}\left(x_{i}\right)}D_{t}\cdot \left|y_{i}-H_{t}\left(x_{i}\right)\right|}{\sum_{i:H_{t}\left(x_{i}\right)}D_{t}}$$

Then $B_{t}$ was computed with (7).

(7) $$B_{t}=\frac{E_{t}}{\left(1-E_{t}\right)},\;0<B_{t}<1$$

The rule of Equation (8) was used to reduce the weights of those data points that were correctly classified by the composite hypothesis $H_{t}$. Furthermore, this lowered the probability of being selected in the following training subset.

(8) $$\alpha_{t+1}\left(i\right)=\alpha_{t}\left(i\right)\cdot \{\begin{array}{cc} B_{t}, & if\;H_{t}\left(x_{i}\right)=y_{i}\\ 1, & otherwise \end{array}$$

**Figure 2:**
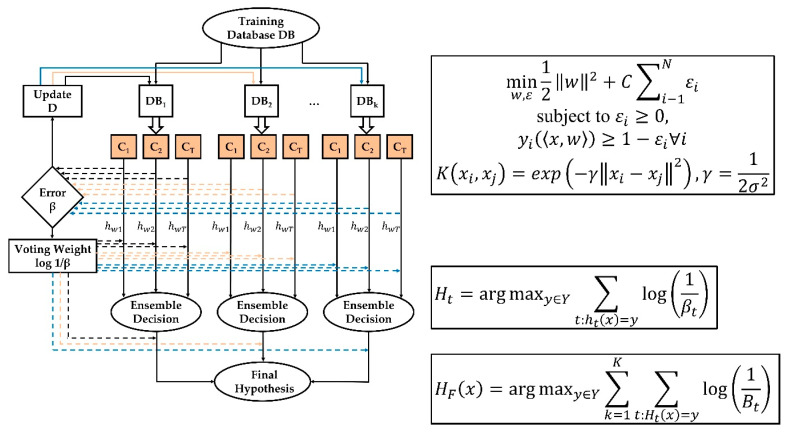
Schematic representation of the incremental learning ensemble classifier (altered from [20]).

The hypothesis $H_{F}$ for the training subset and the subset of features could be obtained by combining all hypotheses that had been generated so far using the weighted majority voting rule (see Figure 2).

(9) $$H_{F}\left(x\right)=\arg\max_{y\in Y}\overset{K}{\underset{k=1}{\sum }}\underset{t:H_{t}\left(x\right)=y}{\sum }\log\left(\frac{1}{B_{t}}\right)$$

### 2.4. Evaluation

The water spectrum was subtracted from each sample’s spectrum. Then, 454 main features from intervals of the spectrum were used to evaluate the data. The intervals contained the spectrum’s descriptive peaks, which were between the Raman shifts 850–910, 1010–1130, 1410–1510, and 2840–3010 cm^−1^. These characteristics corresponded to the value of the derivative and the integral in each interval.

The following methodology was implemented to obtain an objective evaluation. The data set was divided into three subsets: training, validation, and test. The validation subset aimed to make a fair estimation of performance, independency of the test data, and to pick optimal parameters, which in turn provided a more generalized solution. We implemented a nested cross-validation (CV) method for unbiased estimation of prediction error in an independent test data. Leave-one-out (LOO) was used to evaluate the classifier, i.e., nine experiments were used for training and validation and one experiment for independent testing. To estimate the classifier’s unknown tuning parameters, tenfold CV was used with data points from the nine experiments selected for training and validation. The data points were randomly permuted and divided in ten parts, one part for the validation and nine for training. Statistical measures such as the sensitivity true positive results (TPR) and precision were also computed (see Equation (10)). A thresholding procedure was used to design a binary classification. The threshold $T_{h}$ was selected as the concentration level such that a particular concentration was higher than the threshold concentration if the probability $p\left(\frac{y_{i}}{x_{i}}\right)\geq T_{h}$ else it was classified as lower concentration than required, and a ground truth table based on this idea was generated for every concentration. The use of TPR and Positive Predictive Value (PPV) provided additional information about the classifier’s performance and could be used to compare results obtained with other methods. Additional parameters like the PPV and TPR were also calculated as follows:

(10) $$\mathrm{TPR}=\frac{\mathrm{TP}}{\mathrm{TP}+\mathrm{FN}}$$

(11) $$\mathrm{PPV}=\frac{\mathrm{TP}}{\mathrm{TP}+\mathrm{FP}}$$

TP and TN denote the number of true positive and true negative data points, respectively. FP and FN denote the number of false-positive and false-negative data points, respectively.

The SVM model in The Unscrambler X version 10.4 (Camo Software, Oslo, Norway) was used to compare the algorithm with a standard program. The ground truth table generated using the MATLAB algorithm was used as input data for binary classification. For each concentration, cross-validation was performed in the same way the MATLAB algorithm does. This resulted in the creation of nine models and predictions per concentration.

## 3. Results

**Figure 3:**
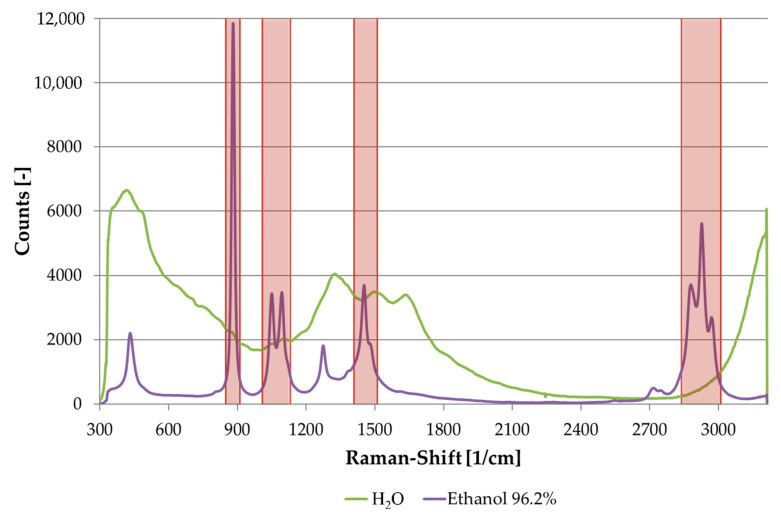
Raman spectra of water and ethanol with evaluated intervals.

Figure 3 shows the resulting Raman spectra of ethanol and water in the complete recorded region. The descriptive peaks for ethanol in between 850–910, 1010–1130, 1410–1510, and 2840–3010 cm^−1^ were used in the algorithm. Additionally, two Raman peaks at 435 and 1275 cm^−1^ were also visible in the ethanol spectrum. Water showed typical bands at 500 cm^−1^ (hydrogen bond), 1640 cm^−1^ (OH bending), and 3100–3600 cm^−1^ (OH stretching). Remaining peaks were derived from the glass beaker.

**Figure 4:**
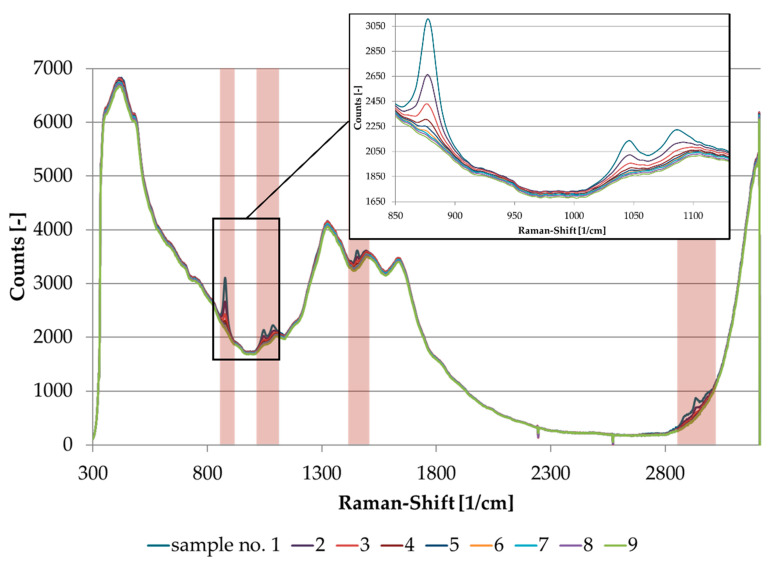
Raman spectra of ethanol dilution series (sample number 1–9).

In Figure 4, the spectral data of the dilution series is displayed. For reasons of clarity, the range in between 850–1130 cm^−1^ was enlarged to show the linear concentration dependency more clearly.

The outputs from the predictions obtained through Unscrambler X must be categorised manually into true positive, true negative, false positive, and false negative to calculate the mean value for accuracy, precision, and recall (sensitivity) for each concentration. The results obtained through Unscrambler X are shown in Table 2.

Table 3 displays the results obtained from the MATLAB algorithm. The different parameters for the performance of the classifier were computed using the algorithm. The training time, as well as the classification time, was calculated for each concentration. The time taken for validation and training was also calculated.

As can be observed from the results obtained, when the concentration is higher, both the methods’ performances were more or less identical. However, as the concentration decreases, the MATLAB algorithm showed better accuracy, precision, and sensitivity. For concentrations from 0.962 to 0.12025, the performance with respect to precision and sensitivity of both methods was almost the same, but the MATLAB algorithm’s accuracy was still higher. As we go to lower concentrations from 0.060125 to 0.007515625, there was a significant difference between the accuracy, precision, and sensitivity from the MATLAB algorithm and Unscrambler X. This shows that the MATLAB algorithm gave better results in terms of accuracy, precision, and sensitivity.

**Figure 5:**
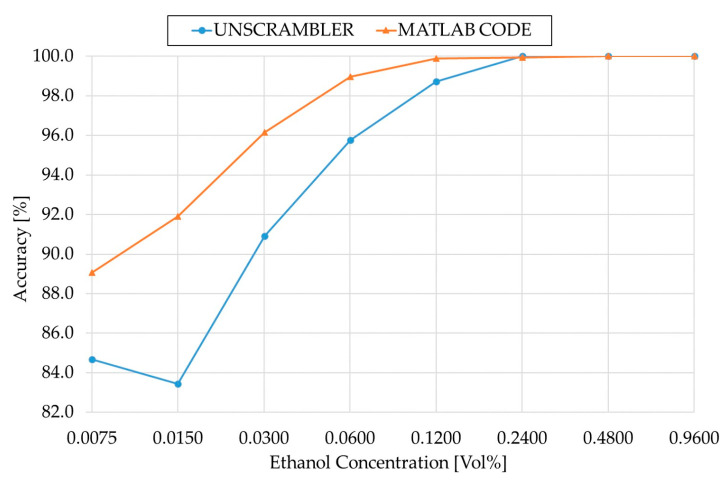
Comparison of the accuracy from The Unscrambler X and the MATLAB code.

In Figure 5, the accuracy from The Unscrambler X and the accuracy from the MATLAB algorithm are plotted over concentration. For concentrations from sample number 1–3, the Unscrambler X models with the MATLAB parameters showed a significantly lower accuracy, while the MATLAB algorithm and the Unscrambler X model with the new parameters showed an accuracy of 100%. This implied that the Unscrambler X and MATLAB work with different parameters that cannot be taken from each other to create the same results. For calculated ethanol concentrations lower than 0.481 Vol% (sample number 3), the MATLAB algorithm generated higher accuracy and displayed slighter decrease in accuracy over the samples. At a calculated ethanol concentration of 0.0300625 Vol% (sample number 7), the MATLAB algorithm had the highest difference in accuracy compared to the Unscrambler model with approximately 8%.

## 4. Discussion

To solve the problem of unlearning in models used in PAT processes, pattern recognition systems that can be better adapted, scalable, and given the ability to learn different data distributions dynamically should be used, but are not available. The method for incremental learning algorithm that uses an ensemble algorithm for classification in a rectification process of ethanol is a possible solution for unlearning. It can learn additional information as soon as new data are available without unlearning the previously acquired knowledge and prevents the unlearning of old Raman spectroscopic data. The system is continuously updated with the current data and significantly increases robustness and accuracy without the loss of any data.

The main advantage of the proposed method over the standard methods available, like Unscrambler X, was that the computation time was less, accuracy was higher, and manual intervention was not involved. The disadvantage in the SVM model created using Unscrambler X was that the data had to be prepared manually, and for each concentration, a model had to be created. The proposed method works without manual intervention saving the users a lot of time and effort. Additionally, a Grid Search had to be performed for each concentration in Unscrambler X. This resulted in a total of 81 models that had to be evaluated manually. Hence, the total time to calculate the accuracy for all concentrations depended on the operator’s efficiency and speed.

The algorithm of the developed new model’s self-learning follow-up enabled the computing time (batch processing, parallel execution, and distributed data) to be reduced. Additionally, the accuracy and the detection sensitivity of the measuring devices that are used can be increased. In this paper, the spectrum range peaks were selected and used, but the algorithm’s application can be extended to any number of peaks and any range in the spectrum that suits the application. The algorithm can be adapted for different data and applications where continuous monitoring is required with the addition of new data. The algorithm performs better than standard methods until the concentration is above 0.0075.

Further investigations need to be performed using different ranges of spectroscopic data and with different chemical processes. The method presented further increases the robustness against outliers or poorly representative data, which do occur in the measured values obtained during operation determined by the system’s operating personnel. The fast availability of measurement data increases the system flexibility and thus the constant optimization to changing demands. To the best of our knowledge, the present work is the first to apply the incremental learning SVM model in Raman spectroscopic data used in various chemical processes where continuous monitoring is required.

## Figures and Tables

**Table 1:** Dedicated samples numbers with calculated ethanol concentration.

Sample Number	Volume H_2_O/mL	Volume EtOH/mL	Calculated EtOH Concentration/Vol%
0	50.000000000	0.000000000	0.000000000
1	49.500000000	0.500000000	0.962000000
2	49.750000000	0.250000000	0.481000000
3	49.875000000	0.125000000	0.240500000
4	49.937500000	0.062500000	0.120250000
5	49.968750000	0.031250000	0.060125000
6	49.984375000	0.015625000	0.030062500
7	49.992187500	0.007812500	0.015031250
8	49.996093750	0.003906250	0.007515625
9	49.998046875	0.001953125	0.003757813

**Table 2:** Calculated mean value for accuracy, precision, recall/sensitivity, and computation time for each concentration with the c-value and gamma obtained from the algorithm using Unscrambler X.

Calculated EtOH Concentration (Vol%)	Accuracy Unscrambler (Unscrambler Parameters) (%)	Precision (-)	Recall/Sensitivity (-)
0.9620	100.0	1.00	1.00
0.4810	100.0	1.00	1.00
0.2405	100.0	1.00	1.00
0.1203	98.7	0.99	0.98
0.0601	95.8	0.95	0.97
0.0301	90.9	0.95	0.90
0.0150	83.4	0.90	0.87
0.0075	84.7	0.90	0.92
0.0038	90.7	0.93	0.98

**Table 3:** Calculated mean value for accuracy, precision, recall/sensitivity, and time required for training and validation for each concentration obtained from the MATLAB algorithm.

Calculated EtOH Concentration (Vol%)	Accuracy MATLAB Algorithm (MATLAB Algorithm Parameters) (%)	Precision (-)	Recall/ Sensitivity (-)	Training and Validation Time (s)
0.9620	100.0	1.00	1.00	14.5748
0.4810	100.0	1.00	1.00	17.8897
0.2405	99.9	0.99	1.00	43.3492
0.1203	99.9	0.98	0.98	40.5498
0.0601	99.0	0.97	0.97	148.2467
0.0301	96.2	0.96	0.95	225.5823
0.0150	91.9	0.97	0.93	140.3069
0.0075	89.1	0.98	0.93	92.7786
0.0038	83.7	0.99	0.94	48.4829

## Data Availability

Restrictions apply to the availability of these data. Data was obtained from José Fernando García Molina’s thesis and is available from the authors with the permission of García Molina.
